# Crystal structures of CDC21-1 inteins from hyperthermophilic archaea reveal the selection mechanism for the highly conserved homing endonuclease insertion site

**DOI:** 10.1007/s00792-019-01117-4

**Published:** 2019-07-30

**Authors:** Hannes M. Beyer, Kornelia M. Mikula, Tatiana V. Kudling, Hideo Iwaï

**Affiliations:** Research Program in Structural Biology and Biophysics, Institute of Biotechnology, University of Helsinki, P.O. Box 65, 00014 Helsinki, Finland

**Keywords:** Inteins, Protein splicing, Homing endonuclease, Hyperthermophiles, Horizontal gene transfer

## Abstract

Self-splicing inteins are mobile genetic elements invading host genes via nested homing endonuclease (HEN) domains. All HEN domains residing within inteins are inserted at a highly conserved insertion site. A purifying selection mechanism directing the location of the HEN insertion site has not yet been identified. In this work, we solved the three-dimensional crystal structures of two inteins inserted in the cell division control protein 21 of the hyperthermophilic archaea *Pyrococcus abyssi* and *Pyrococcus horikoshii*. A comparison between the structures provides the structural basis for the thermo-stabilization mechanism of inteins that have lost the HEN domain during evolution. The presence of an entire extein domain in the intein structure from *Pyrococcus horikoshii* suggests the selection mechanism for the highly conserved HEN insertion point.

**Electronic supplementary material:**

The online version of this article (10.1007/s00792-019-01117-4) contains supplementary material, which is available to authorized users.

## Introduction

Protein splicing is catalyzed by intervening protein sequences termed inteins (Paulus 2000; Perler et al. 1994). Self-splicing inteins can be divided into three different groups based on their primary structures (Paulus 2000; Gogarten et al. 2002). The first group encompasses canonical inteins bearing a homing endonuclease (HEN) domain that can be one of the various types of HEN domains, including the most common LAGLIDADG family and is either active or degenerated (Barzel et al. 2011). Mini-inteins lacking a HEN domain constitute the second group (Novikova et al. 2016). Naturally occurring mini-inteins generally seem proficient in catalyzing protein splicing, suggesting that protein-splicing HINT (Hedgehog/INTein) and HEN domains are functionally independent of each other. A few mini-inteins are naturally split into two fragments and often regarded as a distinct third group due to their bi-molecular splicing reaction (Wu et al. 1998; Iwai et al. 2006; Dassa et al. 2009). Split inteins catalyze protein splicing in *trans*, thereby ligating two flanking polypeptide chains into one chain and have been used for various synthetic biological and biotechnological applications (Wood and Camarero 2014; Volkmann and Iwaï 2010).

Inteins are particularly prevalent in archaea as they were found in nearly half of all sequenced archaeal genomes, whereas only one percent of eukaryotic genomes contain inteins; these are further limited to unicellular organisms (Novikova et al. 2016). It is believed that inteins have spread across taxa via horizontal gene transfer (HGT) facilitated by the nested HEN domains. Once intein genes are fixed in an organism, nested HEN domains in inteins are constantly facing degeneration due to the lack of activity-driven selection (Gogarten et al. 2002; Gogarten and Hilario 2006). Thus, one might assume that mini-inteins have lost their HEN domains during evolution. Some inteins have developed a mutualism between HINT and HEN domains, thereby avoiding the complete loss of the HEN domain (Iwaï et al. 2017). Comparison of structure and function among various inteins could shed light on how each intein has spread and evolved. In the genomes of *Pyrococcus abyssi* and *Pyrococcus horikoshii* OT3, 14 inteins have been reported (Pietrokovski 2001). It remains elusive why some archaeal genomes have accumulated dozens of intein insertions. The investigation of the intein profile of such organisms could provide new insights into the relationship between different inteins and their host organisms and unveil details on the history of horizontal gene transfer events.

As the first step toward understanding the spread and evolution of inteins and cross-talks within the “inteinome”, we determined the crystal structures of mini-inteins inserted at the same insertion site within the putative cell division control protein 21 (Cdc21) from the hyperthermophilic archaea, *Pyrococcus abyssi* and *Pyrococcus horikoshii* OT3.

## Results

The cell division control protein 21 (Cdc21), belonging to the MCM (mini-chromosome maintenance) protein family, is one of the proteins invaded by several inteins (Pietrokovski 2001). Particularly hyperthermophiles such as *Pyrococcus abyssi* and *Pyrococcus horikoshii* contain multiple inteins inserted into the same Cdc21 protein (Pietrokovski 2001). While some thermophilic inteins like the TFIIB intein from *Methanocaldococcus jannaschii* (*Mja*TFIIB intein) require the HEN domain or a long linker at the HEN insertion site for protein-splicing activity, mini-inteins from *Pyrococcus* are capable of protein splicing even at 37 °C (Fig. S1) (Iwaï et al. 2017; Ellilä et al. 2011). To understand the structural basis of the difference between inteins from hyperthermophiles, we investigated three-dimensional structures of mini-inteins from the *Pyrococcus* genus.

### Structure of *Pab*CDC21-1 intein

**Fig. 1:**
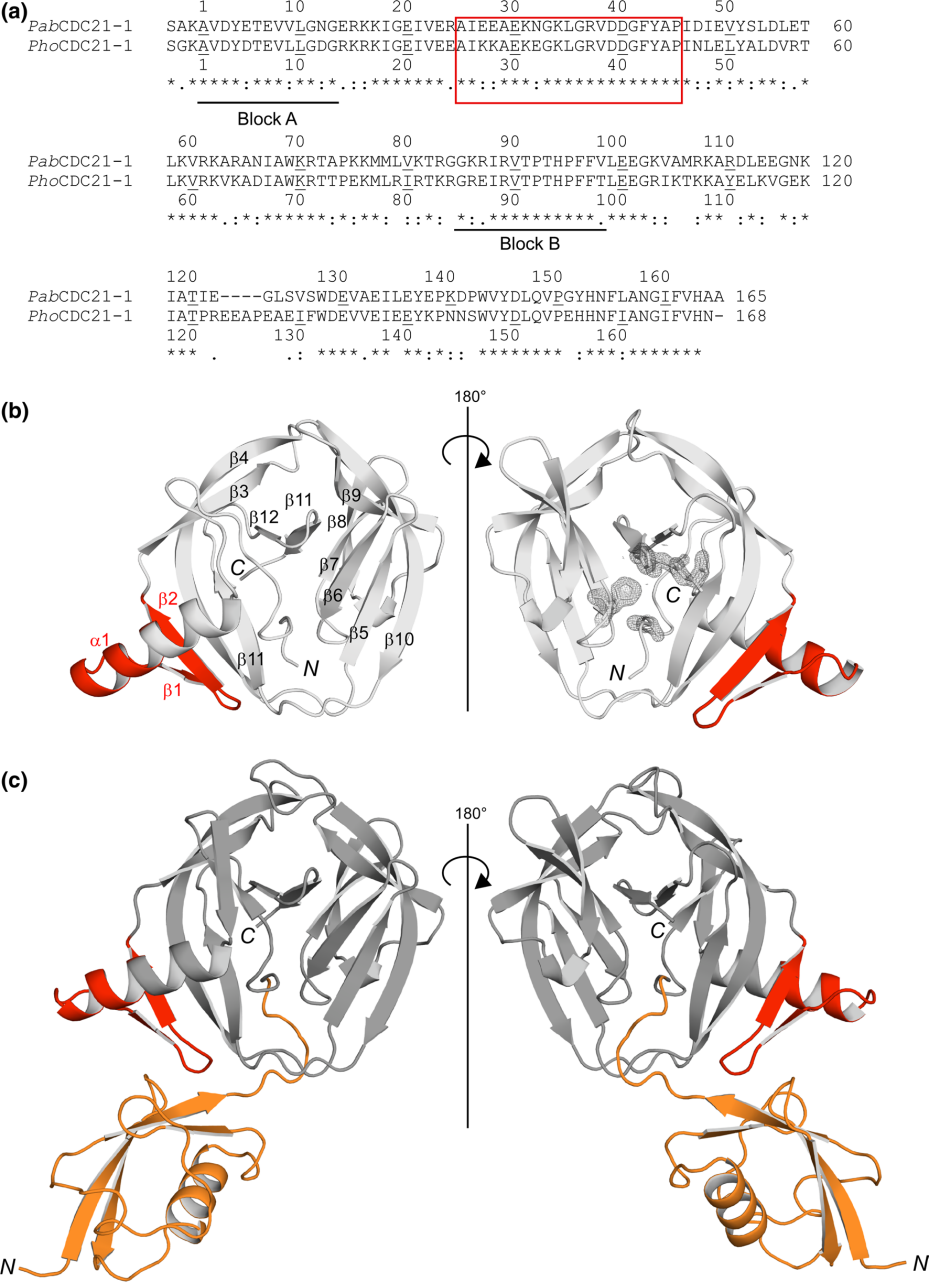
Structure of the *Pab* and *Pho* CDC21-1 inteins. **a** Primary structure comparison between the *Pab*CDC21-1 and *Pho*CDC21-1 inteins. Conserved sequence Blocks A and B are indicated. **b** The crystal structure of the *Pab*CDC21-1 intein. The final electron density map, contoured at 1.3 σ-level, is shown for the active site residues. **c** The crystal structure of the *Pho*CDC21-1 intein together with the fused extein (SUMO domain). The SUMO N-extein domain is colored in orange. **a–c** The insertion typical for thermophilic inteins is colored in red

The hypothetical Cdc21 protein from *Pyrococcus abyssi* (Uniprot: Q9UYR7) harbors the two inteins CDC21-1 (*Pab*CDC21-1) and CDC21-2 (*Pab*CDC21-2) consisting of 164 and 268 residues, respectively. Both are mini-inteins lacking HEN domains. The *Pab*CDC21-1 intein is inserted at the presumable P-loop ATP/GTP-binding motif; while, the *Pab*CDC21-2 intein locates 27 residues downstream of the *Pab*CDC21-1 intein insertion site. According to our experience, inteins with shorter loops at the HEN insertion site tend to crystallize and diffract better than inteins with longer loops (Oeemig et al. 2012). Indeed, we could obtain diffracting crystals of a variant of the *Pab*CDC21-1 intein carrying mutations at the N- and C-terminal catalytic residues (C1A, and N164A) together with three N- and one C-extein residue (Ser-Ala-Lys and Ala, respectively). We solved the three-dimensional structure of *Pab*CDC21-1 intein at 1.60-Å resolution by molecular replacement (Fig. 1). The structure of the *Pab*CDC21-1 intein revealed the HINT fold characteristic for inteins from thermophilic organisms (Aranko et al. 2014a). In contrast to most mesophilic inteins, thermophilic inteins often contain an additional insertion of two β-strands connected with a helix extension between Blocks A and B, approximately 25 residues distant from the N-terminus (Fig. 1a–c) (Aranko et al. 2014a). Because this additional sequence insertion is commonly found among inteins from thermophiles, one could speculate about a possible role in structure stabilization at elevated temperatures, or a functional role specific for thermophilic inteins. The presence of N- and C-extein residues due to the active site mutations allowed us to analyze the conformation of the *Pab*CDC21-1 intein by measuring the distance between the N-scissile peptide and the side chain of the +1 residue (Cα atom of Ala). A large distance of about 8 Å suggests an open conformation as it has been observed in most intein structures containing extein residues (Oeemig et al. 2012; Mizutani et al. 2002).

### Structure of *Pho*CDC21-1 intein

*Pyrococcus horikoshii* OT3 also contains the CDC21-1 intein (*Pho*CDC21-1) at the same site as the *Pab*CDC21-1 intein within the P-loop region (UniProt: O58310). The *Pho*CDC21-1 intein consists of 168 residues, which is four residues longer than the *Pab*CDC21-1 intein. Both share a pairwise sequence identity of 64% (108/168). We introduced the C1A mutation into the *Pho*CDC21-1 intein without additional C-extein sequence and fused it to an N-terminal H_6_-SUMO domain (yeast SMT3) for convenient purification (Guerrero et al. 2015). Typically, three residues between the cleavage site of the Ubiquitin-like specific protease 1 (Ulp1) and the first residue of an intein are sufficient for proteolytic cleavage of the SUMO purification tag (Guerrero et al. 2015). Interestingly, the three-residue “SGK” linker used between the H_6_-SUMO and *Pho*CDC21-1 intein in the fusion protein was too short for efficient cleavage by Ulp1. However, a very similar linker sequence was sufficient for the proteolytic removal of the H_6_-SUMO fusion from the *Pab*CDC21-1 intein. Thus, we performed crystallization trials with the H_6_-SUMO-*Pho*CDC21-1 fusion protein without removal of H_6_-SUMO domain and were successful in obtaining diffracting crystals. We were able to solve the structure at 2.65-Å resolution by molecular replacement using the coordinates of the *Pab*CDC21-1 intein and the SUMO domain (PDB: 1EUV) as search models. Overall, the crystal structure of the *Pho*CDC21-1 intein is very similar to that of *Pab*CDC21-1 but includes the SUMO domain as N-extein (Fig. 1b–c).

### Comparison between *Pab* and *Pho* CDC21-1 inteins

**Fig. 2:**
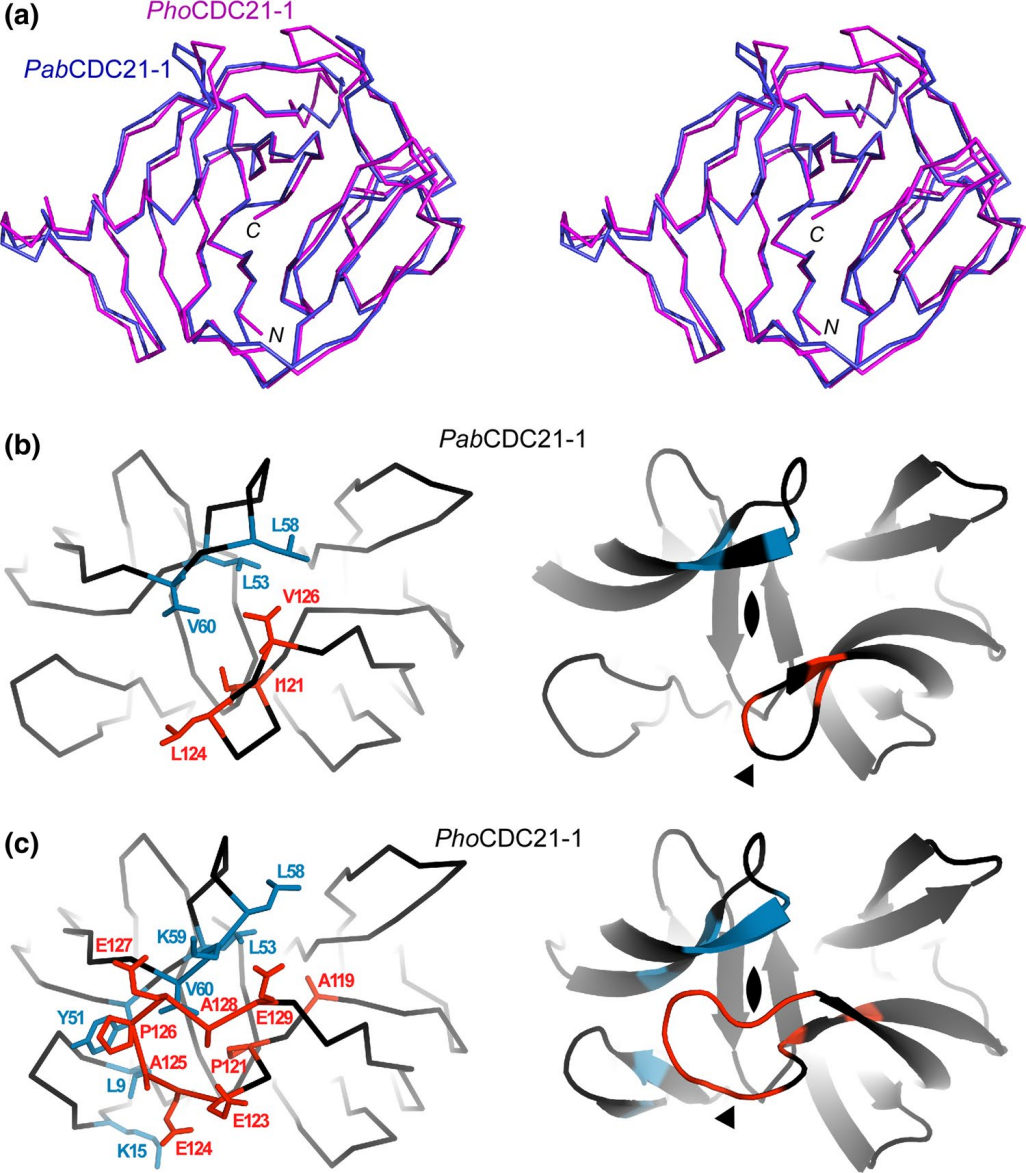
Comparison between *Pab*CDC21-1 and *Pho*CDC21-1 inteins. **a** Stereoview of the superposition of the two crystal structures, *Pab*CDC21-1 intein (light blue) and *Pho*CDC21-1 intein (magenta). **b** Schematic illustrations of the intramolecular interactions between the N35 (blue) and C35 (red) loops in the *Pab*CDC21-1 intein. **c** Schematic illustrations of the intramolecular interactions between the N35 (blue) and C35 (red) loops in the *Pho*CDC21-1 intein. **b–c** An arrowhead indicates the HEN insertion site

**Fig. 3:**
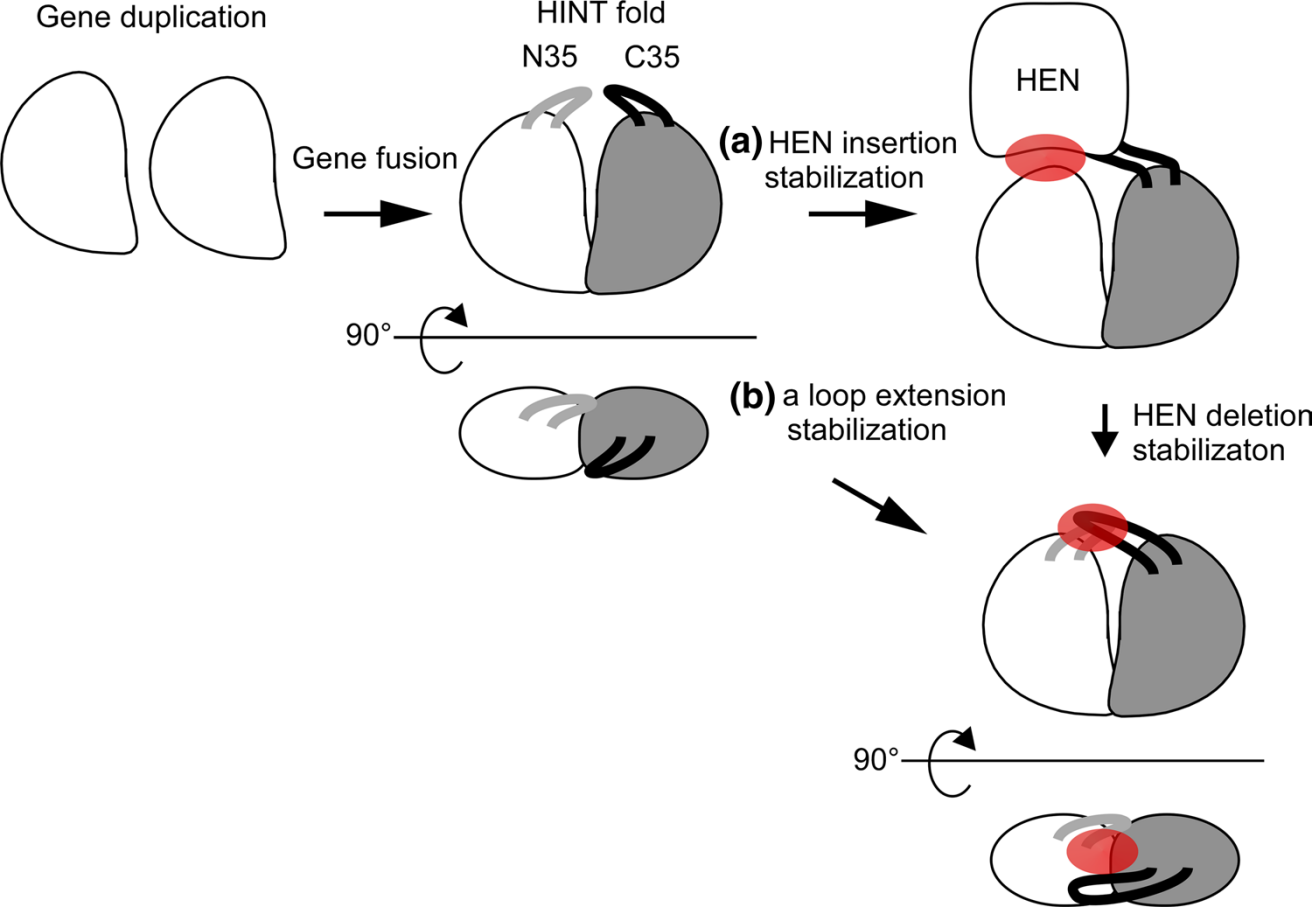
Cartoon model of the stabilization mechanisms in inteins from thermophilic organisms. **a** The HINT fold has presumably emerged from gene duplication and fusion events. Stabilization by fixing the two subdomain orientations with the nested HEN domain (Iwaï et al. 2017). **b** The loop at the HEN insertion site adopts the stabilization function of the two HINT subdomains to countervail thermal motions at the elevated temperature in the case of hyperthermophilic CDC21 mini-inteins. The stabilizing interactions are highlighted in red

The HINT fold of the *Pab* and *Pho* CDC21-1 inteins is almost identical with an RMSD of 1.1 Å for 162 pairs of aligned Cα atoms, as expected from the high sequence identity of 64% (Fig. 2a). The main difference between the two structures is a four-residue loop extension located at the canonical HEN insertion site of the *Pho*CDC21-1 intein (Fig. 2b, c). The HINT domain subdivides into two subdomains, which are presumably the result of gene duplication and fusion, resulting in a C2-symmetry. *Pab*CDC21-1 has an improved C2-symmetry interface with the loop regions at the C35 and N35 sites with hydrophobic residues L53, L58, and V60 for the N-terminal, and I121, L124, and V126 for the C-terminal subdomain (the C35 and N35 numbering for the split sites is based on the *Npu*DnaE intein as described in Aranko et al. (2014a) for convenience) (Fig. 2b). The 4-residue loop extension in the *Pho*CDC21-1 intein creates additional interactions, presumably stabilizing the two subdomains further. The additional interactions are made by hydrophobic and charged residues (Fig. 2c). The presence of stabilizing interactions within the extended loop is in line with previous observations in other thermophilic inteins, the *Mja* and *Mvu *TFIIB inteins, which showed that the artificial deletion of the HEN domain caused a significant deterioration of the protein splicing activity. However, introducing longer loops partially restored the splicing activity by compensating the removal of the HEN domain for the splicing activity (Fig. 3) (Iwaï et al. 2017). Similar results were also obtained for non-thermophilic inteins when HEN domains were removed (Hiraga et al. 2005). These observations suggest that the proper arrangement of the two subdomains in the HINT domain is critical for efficient protein splicing catalysis. Utilizing the loop at the C35 split site could be a general strategy of inteins to stabilize active conformations and might be of special importance for thermophilic inteins.

### Unexpected interactions between the N-extein and *Pho*CDC21-1 intein

**Fig. 4:**
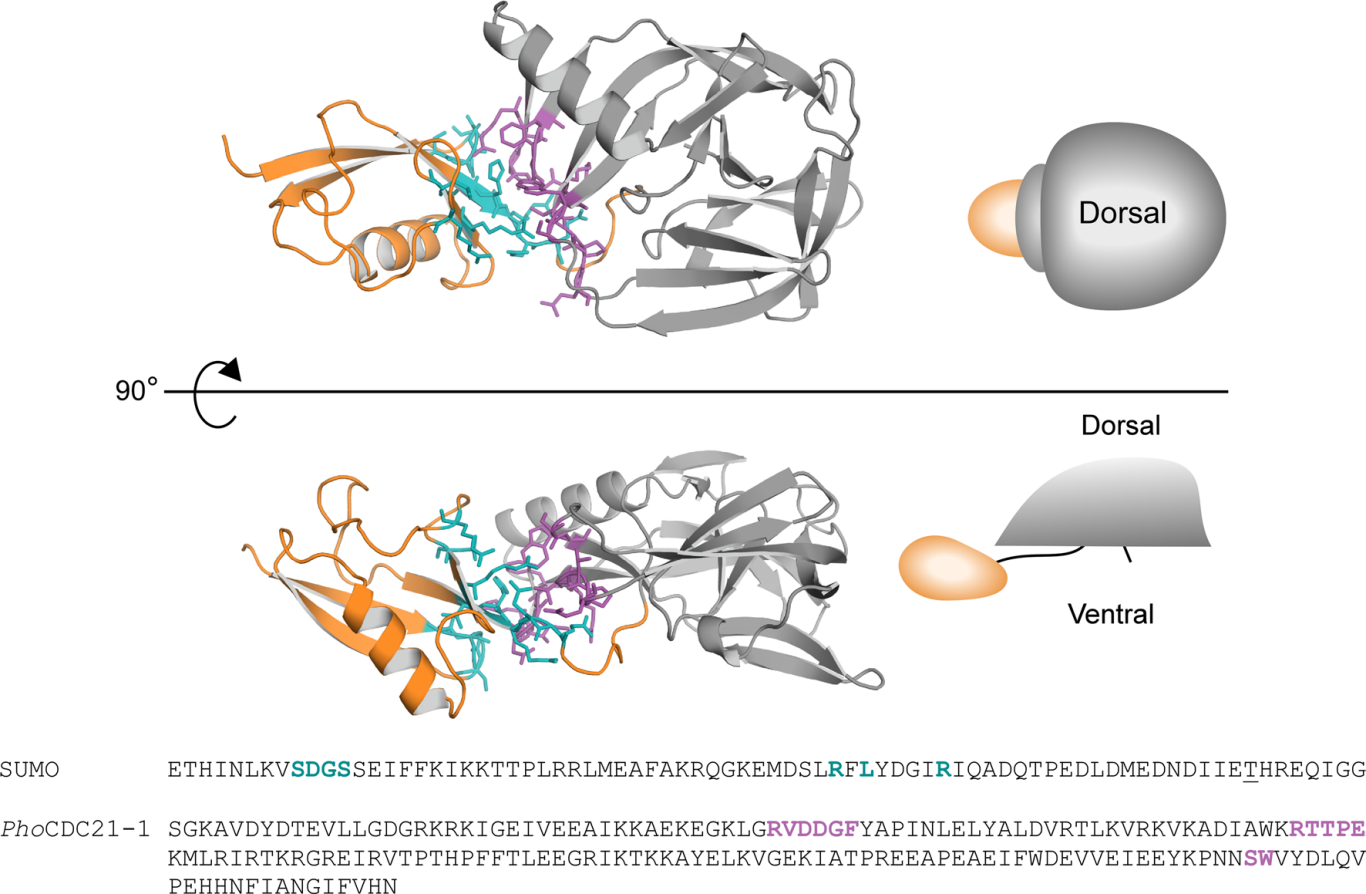
Interactions between the *Pho*CDC21-1 intein and the N-extein (SUMO). The upper panel shows the view from the dorsal side of a horseshoe-crab shape. The lower panel shows the side-view. The N- and C-termini of the intein locate at the ventral side. The inserted β-strand and extended α-helix commonly found in inteins from hyperthermophiles face to the dorsal side. Residues contributing to the interaction between the HINT domain (gray) and the N-terminal SUMO extein (orange) are shown as teal and magenta sticks, respectively. The residues are highlighted in the primary sequences below. An Ala to Thr mutation in the SUMO domain in the used vector is underlined

Despite very similar structures and sequences of the *Pab*CDC21-1 and *Pho*CDC21-1 inteins, we could not proteolytically remove the fused SUMO domain from the *Pho*CDC21-1 intein using Ulp1 protease. Therefore, we crystallized the fusion protein. We observed clear electron densities for the entire SUMO domain in addition to the *Pho*CDC21-1 intein, except for the 30 N-terminal residues containing the His-tag. To our knowledge, this is the first intein structure with an entire extein domain in addition to the junction regions. Of interest are interactions that we observed between the SUMO domain and the *Pho*CDC21-1 intein (Fig. 4). These interactions were presumably absent in the H_6_-SUMO–*Pab*CDC21-1 intein, leading to efficient digestion by Ulp1 protease. The interacting residues at the SUMO/intein interface locate mainly on one subdomain of the *Pho*CDC21-1 intein (residues 37–42, 71–75, and 146–147). The location also coincides with the β-turn of the extension found in inteins from hyperthermophiles (Figs. 1 and 4). On the SUMO domain, interacting residues mainly cover the C-terminal side. Because the amino-acid sequences at the interfaces are similar between the *Pho* and *Pab* CDC21-1 inteins, these interactions could be rather weak and depend on the covalent linkage of the SUMO domain to the *Pho*CDC21-1 intein as well as on the crystal packing. The poorer X-ray diffraction obtained for the crystal of the H_6_-SUMO-*Pho*CDC21-1 intein fusion could also be explained by the relatively large deviations of the SUMO structure with respect to the *Pho*CDC21-1 intein structure presumably because of the weak interactions. We previously observed that *cis*-splicing of the *Pho*CDC21-1 intein strongly depends on the fused exteins when artificial exteins are used—even while maintaining identical junction sequences (Ellilä et al. 2011). The observed extein–intein interactions confirm the structural basis of this extein-context dependency in addition to the well-known junction-sequence dependency common among inteins (Iwai et al. 2006; Oeemig et al. 2012; Aranko et al. 2009; Topilina et al. 2015).

## Discussion

Many inteins contain an active or degenerated homing endonuclease domain, which has presumably played (or still plays) an important role in the propagation of intein genes via horizontal gene transfer (Gogarten et al. 2002; Barzel et al. 2011; Okuda et al. 2003; Koufopanou and Burt 2005). Even though many mini-inteins including *Pab*CDC21-1 and *Pho*CDC21-1 are capable of protein splicing and hence independent of HEN domains for protein splicing, some inteins have developed a mutualism between the HEN and HINT domains. Examples include the TFIIB inteins from *Methanococcus jannaschii* and *Methanocaldococcus vulcanius*, both of which require the HEN domain for efficient protein splicing activity (Iwaï et al. 2017). The endonuclease domain nested in the *Mja*TFIIB intein is a degenerated HEN domain but presumably plays a role in fixing the orientation of the two pseudo-domains of the HINT fold, because deletion of the HEN domain caused a uniquely open conformation (Iwaï et al. 2017). The crystal structures of the *Pab*CDC21-1 and *Pho*CDC21-1 inteins showed that both mini-inteins do not have large flexible loop insertions at the HEN insertion site, unlike the *Mja*TFIIB intein. The structure of the *Pab*CDC21-1 intein revealed extensive hydrophobic interactions between the C2-symmetry-related N35 and C35 loops, increasing the subdomain interactions at the elevated temperature optimal for the growth of *Pyrococcus abyssi* (Fig. 2b). The four-residue insertion at the HEN insertion site of the *Pho*CDC21-1 intein further enhances the intramolecular stabilization between the two subdomains compared with the *Pab*CDC21-1 intein (Fig. 2c). The extended loop at the HEN insertion site bridges the two subdomains via additional hydrophobic and electrostatic interactions which likely substitute the stabilization role of the HEN domain in inteins like *Mja*TFIIB (Fig. 3). The precise orientation of the subdomains stabilized by the loops might be of particular importance at elevated temperatures where hyperthermophiles inhabit.

**Fig. 5:**
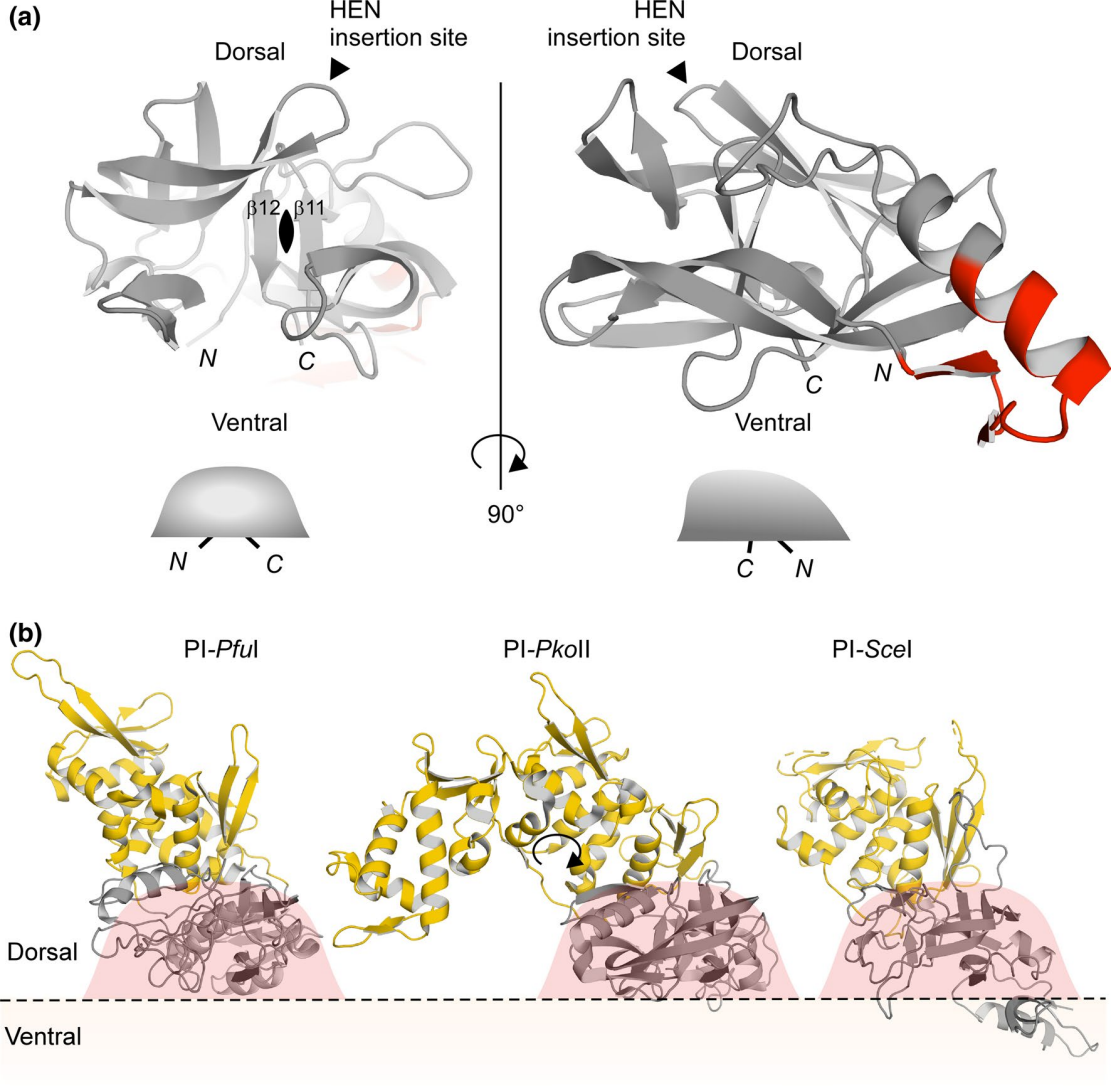
Structural features of the HEN insertion site on the horseshoe-crab shape of inteins. **a** A side-view of the *Pab*CDC21-1 intein with the C2-symmetry around the HEN insertion site. The HEN insertion locates on the dorsal side of the horseshoe-crab shape. The thermophilic insertion is colored in red. **b** The locations of the HEN domains in the three crystal structures of PI-*Pfu*I (PDB: 1DQ3), PI-*Pko*II (PDB: 2CW8), and PI-*Sce*I (PDB: 1JVA) (Mizutani et al. 2002; Ichiyanagi et al. 2000; Matsumura et al. 2006). The HEN and HINT domains are colored in yellow and gray, respectively. The horseshoe-crab shape is indicated as red shape on top of the HINT domains

It has been puzzling why the HEN insertion site (C35 site) is extremely conserved among inteins. There is no obvious purifying selection of the HEN domains to exclusively locate at that specific position (Gogarten and Hilario 2006). If a free-standing endonuclease domain randomly invaded into inteins, inteins should exist in which HEN domains have inserted at other sites than the C35 site (Iwaï et al. 2017). For example, the C2-symmetry-related N35 site could be an equally suited insertion point. The structures of the hyperthermophilic CDC21-1 inteins might provide a hint for the high conservation of the HEN insertion site. The HINT fold has often been referred to as a flat horseshoe shape (Hall et al. 1997). Because of the C2 symmetry in the HINT fold, one can define an upper (dorsal) and lower (ventral) side (Figs. 4 and 5). The HINT fold, thus, rather resembles a horseshoe-crab shape with a carapace than a horseshoe shape due to the C2-symmetry (Fig. 4). The ventral side is where the splicing reaction takes place and the N- and C-termini of inteins locate (Figs. 4 and 5). In this architecture, the HEN insertion site locates on the dorsal side (Fig. 5a). The C2-symmetry-related site (N35) of the HEN insertion site points toward the ventral side of the intein structure. Any large insertion at the N35 site would clash with the extein region (host protein). We believe that this steric incompatibility with the host protein sequences (exteins) defines the conservation of the C35 HEN insertion site and restricts random insertions at other positions, including the N35 site. Indeed, all crystal structures of inteins with HEN domains (PI-*Pfu*I, PI-*Pko*II, and PI-*Sce*I) have open space below the ventral side of the HINT fold, providing room for their insertion into host proteins (Fig. 5b) (Mizutani et al. 2002; Ichiyanagi et al. 2000; Matsumura et al. 2006). The protein size of self-splicing inteins widely ranges from 123 to > 1000 residues, presumably because of repeated invasion and degeneration events of homing endonuclease domains (Novikova et al. 2016). Further investigations of the “inteinome” by elucidating intein structures will shed light on the historical invasion, fixation, and adaptation of inteins as molecular fossils of the intein emergence and evolution.

## Methods

### Cloning of *Pab*CDC21-1 intein

The gene encoding the *Pab*CDC21-1 intein was amplified from genomic DNA of *Pyrococcus abyssi,* DSM-25543, using the two oligonucleotides I725: 5′-AAAGGATCCGCGAAATGCGTCGATTATGAAACTG and I726: 5′-AAAGGTACCAAGTTGGCTGTTGTGAACGAAGATC. The PCR product was cloned between *Bam*HI/*Kpn*I of pHYRSF53 (Addgene #64696), resulting in plasmid pBHRSF151. The plasmid pBHRSF151 encodes a *cis*-splicing precursor with a H_6_-SUMO domain (yeast SMT3) and Chitin-Binding Domain (CBD) as N- and C-exteins, respectively. For structural studies, the C1A and N164A mutations were introduced into the *Pab*CDC21-1 intein, and the C-terminal extein at the +1 position was replaced with a single Ala residue (T + 1A). The fusion protein was constructed by cloning the PCR product amplified from pBHRSF151 using the two oligonucleotides J303: 5′-AAAGGATCCGCGAAAGCCGTCGATTATGAAACTG and J304: 5′-GCGGTACCTTATGCAGCGTGAACGAAGATCCCATT between *Bam*HI and *Kpn*I sites of pHYRSF53, resulting in plasmid pBHRSF399 encoding the H_6_-SUMO-fused *Pab*CDC21-1 (C1A, N164A, T + 1A) intein.

### Cloning of *Pho*CDC21-1 intein

For the structural investigation, the *Pho*CDC21-1 intein with the C1A mutation was constructed as an N-terminally His-tagged SUMO fusion similar to the *Pab*CDC21-1 intein variant described above. The gene of the *Pho*CDC21-1 intein was amplified from the previously published *cis*-splicing vector pSKDuet21 (Addgene #41695) in which the intein is flanked by two B1 domains of IgG binding protein G (GB1) with N-terminal His tag (Ellilä et al. 2011). For PCR amplification, the two oligonucleotides HK937: 5′-AGGATCCGGTAAGGCCGTTGACTACGATACAG and HK938: 5′-TTGGTACCTTAATTGTGCACGAATATTCCG were used. The PCR product was cloned into pHYRSF53-36 using the restriction enzymes *Bam*HI and *Kpn*I, resulting in pCARSF52 (Guerrero et al. 2015; Aranko et al. 2009).

### Production and purification of *Pab* and *Pho* mini-inteins

The *Pab*CDC21-1 and *Pho*CDC21-1 inteins were produced by expression of the above-described plasmids pBHRSF399 and pCARSF52 in *E. coli* strain T7 Express (New England Biolabs, Ipswich, USA). pCARSF52 was co-transformed with plasmid pRARE (Merck Millipore, Darmstadt, Germany) to account for rare codons. The transformed cells were grown at 37 °C in 0.5 L and 2 L LB expression cultures supplemented with 25 μg mL^−1^ kanamycin for the expression of *Pab*CDC21-1 and *Pho*CDC21-1 inteins, respectively. Additionally, 5 μg mL^−1^ chloramphenicol was added for the plasmid pRARE for the expression of *Pho*CDC21-1 intein. The cultures were induced with a final concentration of 1 mM isopropyl-β-d-thiogalactoside (IPTG) for 3 h when OD_600_ reached 0.6. The induced cells were harvested by centrifugation at 4700 × *g* for 10 min, 4 °C and lyzed in 20 mL Buffer A (50 mM sodium phosphate, pH 8.0, 300 mM NaCl) using continuous passaging through an EmulsiFlex-C3 homogenizer (Avestin Inc, Ottawa, Canada) at 15,000 psi for 10 min, 4 °C. Lysates were cleared by centrifugation at 38,000 ×*g* for 60 min, 4 °C. The *Pab*CDC21-1 intein was purified using 5 mL HisTrap HP columns (GE Healthcare, Chicago, USA) as previously described, including the removal of the N-terminal H_6_-SUMO fusion domain (Guerrero et al. 2015). For the *Pho*CDC21-1 intein, the second purification step involving proteolytic digestion with the SUMO-specific protease Ulp1 was omitted. Proteins were dialyzed against deionized water and concentrated using Macrosep^®^ Advance Centrifugal Devices 10 K MWCO (PALL Corporation, New York, USA).

### Crystallization of *Pab* and *Pho* mini-inteins

A 316 μM solution (6 mg/mL) of *Pab*CDC21-1 intein (C1A, N164A, T + 1A) and 1.24 mM solution (40 mg/mL) of H_6_-SUMO-*Pho*CDC21-1 intein (C1A) were used for crystallization trials. Drops of 200 nl (100 nl concentrated protein and 100 nl of mother liquor) were set up in 96-well MRC (Molecular Dimensions, Suffolk, UK) crystallization plates using a Mosquito LCP^®^ (TTP Labtech, Melbourn, UK). Diffracting crystals were obtained with mother liquor compositions of 100 mM sodium acetate pH 4.6, 30% (*w*/*v*) polyethylene glycol 4000, and 200 mM ammonium acetate for the *Pab*CDC21-1 intein, and 1.8 M tri-ammonium citrate pH 7 for the *Pho*CDC21-1 intein. 20% glycerol was added for the latter on top of the drop, which served as a cryoprotectant when freezing crystals in liquid nitrogen.

### Diffraction data collection and processing

*Pab*CDC21-1 and *Pho*CDC21-1 crystals belonged to the space groups 20 (*C*222_1_) and 80 (*I*4_1_), respectively. *Pab*CDC21-1 intein diffraction data were collected on beamline I03 at the Diamond Light Source, Oxfordshire, UK and were subsequently indexed, integrated, and scaled to 1.60-Å resolution using the program XDS (Kabsch 2010; Allan et al. 2015). Diffraction data for the crystal of the *Pho*CDC21-1 mini-intein were collected on beamline I04-1 at Diamond Light Source and were processed to 2.65-Å resolution.

### Structure determination and refinement

The structures of the *Pab*CDC21-1 and *Pho*CDC21-1 inteins were solved by molecular replacement. The search model used for the *Pab*CDC21-1 intein was based on the coordinates of the thermophilic *Tvo*VMA intein (PDB: 4O1S) (Aranko et al. 2014b). The initial solution from Phaser was provided to ARP/WARP for auto-building (McCoy et al. 2007; Langer et al. 2008). The model was built with Coot, followed by rounds of refinement using the software Refmac5 and Phenix (Emsley et al. 2010; Murshudov et al. 2011; Adams et al. 2002). The entire polypeptide chain could be traced into the electron density map without breaks for all 168 residues. The quality of the final structure was validated using the MolProbity webserver (Table 1) (Chen et al. 2010).

**Table 1:** Data collection and structure refinement

Intein	*Pab*CDC21-1 (C1A, N164A, T + 1A)	*Pho*CDC21-1 (C1A)
PDB ID	6RPP	6RPQ
Data collection	DIAMOND I03	DIAMOND I04
Space group	*C* 2 2 2_1_	*I* 4_1_
Cell dimensions		
a, b, c (Å)	75.48, 93.59, 49.75	100.92, 100.92, 91.72
α, β, γ (°)	90.00, 90.00, 90.00	90.00, 90.00, 90.00
Wavelength (Å)	0.9763	0.9159
Resolution (Å)	29.38–1.60 (1.70–1.60)	50.46–2.65 (2.81–2.65)
Total reflections	308,001 (48,151)	184,767 (30,483)
Unique reflections	23,504 (3723)	13,373 (2154)
Completeness (%)	99.9 (99.6)	100.0 (99.9)
*I*/*σ*	24.37 (3.58)	18.23 (1.24)
*R*_meas_^a^	0.059 (0.595)	0.094 (1.679)
CC_1/2_^c^	0.999 (0.929)	0.999 (0.713)
Multiplicity	13.1 (12.9)	13.8 (14.2)
Refinement		
Molecules/au	1	1
Resolution (Å)	29.377–1.603 (1.676–1.603)	50.460–2.654 (2.858–2.654)
Reflections (refinement/*R*_free_)	23,498/1178	13,363/669
*R*_work_/*R*_free_^b^	0.1782/0.2178	0.2023/0.2352
Number of atoms		
Protein	1359	2037
Water	132	0
Ligand	11	0
RMS deviations		
Bond length (Å)	0.014	0.004
Bond angles (°)	1.384	0.887
Ramachandran plot (%)		
Most favored regions	96.99	95.95
Outliers	0.00	0.00
Average B-factors (Å^2^)	29.90	111.49
Protein	28.71	111.49
Water	37.63	–
Clash score	1.81 (99th percentile)	12.24 (95th percentile)
Molprobity score	1.11 (99th percentile)	1.88 (99th percentile)

Numbers in parentheses represent the highest-resolution shell

*au* asymmetric unit

^a^$R_{{{\text{meas}}}} = \Sigma _{h} [n/(n - 1)]^{1/2}\Sigma _{i} {{\left| {I_{i} - \left\langle I \right\rangle } \right|} \mathord{\left/ {\vphantom {{\left| {I_{i} - \left\langle I \right\rangle } \right|} {\Sigma _{h} \Sigma _{i} I_{i} }}} \right. \kern-\nulldelimiterspace} {\Sigma _{h} \Sigma _{i} I_{i} }}$, where *I*_i_ is the observed intensity of the *i*th measurement of reflection *h*, $\left\langle I \right\rangle$ is the average intensity of that reflection obtained from multiple observations, and n is the multiplicity of the reflection

^b^$R = \Sigma {{\left\| {F_{o} \left| - \right|F_{c} } \right\|} \mathord{\left/ {\vphantom {{\left\| {F_{o} \left| - \right|F_{c} } \right\|} {\Sigma \left| {F_{o} } \right.}}} \right. \kern-\nulldelimiterspace} {\Sigma \left| {F_{o} } \right|}}$, where *F*_o_ and *F*_c_ are the observed and calculated structure factors, respectively, calculated for all data. *R*_free_ was defined in Brünger (1991)

^c^CC_1/2_ was defined in Karplus et al. (2012)

For the molecular replacement solution of the *Pho*CDC21-1 intein, the coordinates of the *Pab*CDC21-1 intein and the SUMO domain (PDB: 1EUV) were used as search models. The structure was solved with Phenix, followed by iterative rounds of the model building using ARP/WARP and Coot (McCoy et al. 2007; Langer et al. 2008). The initial 30 residues including the N-terminal His tag lacked sufficient density and were not modeled. The resulting model was rebuilt in place using Phenix and refined with Coot, Refmac5, Phenix, and, PDB-REDO (Emsley et al. 2010; Murshudov et al. 2011; Adams et al. 2002; Joosten et al. 2014). The final model was validated using the MolProbity webserver (Table 1) (Chen et al. 2010).

### *Cis*-splicing analysis

*Cis*-splicing of *Pab*CDC21-1 and *Pho*CDC21-1 inteins was analyzed by expressing the above-described constructs pBHRSF151 and pSKDuet21 with identical conditions as done for the production of samples for crystallization, except that the culture volume was reduced to 5 mL. Culture samples for SDS-PAGE analysis were taken before and 1 and 3 h after induction and cultures were harvested by centrifugation as described above. Cell pellets were resuspended and subsequently lysed in 400 µL B-PER reagent (Thermo Scientific, Waltham, USA) for 15 min under continuous shaking at 1000 rpm. Lysates were cleared by centrifugation at 12,300 × *g* for 5 min. His-tagged splicing products were IMAC purified at room temperature (RT) using Ni-NTA spin columns (Qiagen, Hilden, Germany) according to the protocol of the manufacturer. Proteins were eluted in 100 µL elution buffer (50 mM sodium phosphate, 300 mM NaCl, 250 mM imidazole, pH 8.0) and heated at 60 °C for 1 h before samples were analyzed by SDS-PAGE on 16.5% acrylamide gels and visualized using Coomassie Blue staining.

### Accession numbers

Coordinates and structure factors have been deposited to the Protein Data Bank with accession numbers 6RPP for the *Pab*CDC21-1 intein and 6RPQ for the *Pho*CDC21-1 intein.

## Electronic supplementary material

Below is the link to the electronic supplementary material.

Supplementary material 1 (PDF 1012 kb)

## Abbreviations

CBD: Chitin-binding domain
GB1: B1 domain of IgG binding protein G
HEN: Homing endonuclease
HGT: Horizontal gene transfer
HINT: Hedgehog/INTein
IMAC: Immobilized metal ion affinity chromatography
IPTG: Isopropyl-β-d-1-thiogalactopyranoside
MCM: Mini-chromosome maintenance
*Mja*: *Methanococcus jannaschii*
*Mvu*: *Methanocaldococcus vulcanius* M7
*Pab*: *Pyrococcus abyssi*
PDB: Protein data bank
*Pho*: *Pyrococcus horikoshii* OT3
RMSD: Root mean square deviation
RT: Room temperature
SUMO: Yeast SMT3 domain
Ulp1: Ubiquitin-like-specific protease 1
