# Supplementary material for: Crystal structures of CDC21-1 inteins from hyperthermophilic archaea reveal the selection mechanism for the highly conserved homing endonuclease insertion site
Source: Extremophiles. 2019 Jul 30;23(6):669–79. doi: 10.1007/s00792-019-01117-4 (PMC6801210; doi:10.1007/s00792-019-01117-4)
Supplement: Supplementary file 1 — Supplementary material 1 (PDF 1012 kb) [file 792_2019_1117_MOESM1_ESM.pdf]

Supplementrary data

Crystal structures of CDC21-1 inteins from hyperthermophilic archaea reveal the selection mechanism for the highly conserved homing endonuclease insertion site

Hannes M. Beyer, Kornelia M. Mikula, Tatiana V. Kudling & Hideo Iwaï*

Research Program in Structural Biology and Biophysics, Institute of Biotechnology, HiLIFE, University of Helsinki. P.O. Box 65, Helsinki, FIN-00014, Finland

*To whom correspondence should be addressed.

Phone: +358 2941 59752

Email: hideo.iwai@helsinki.fi

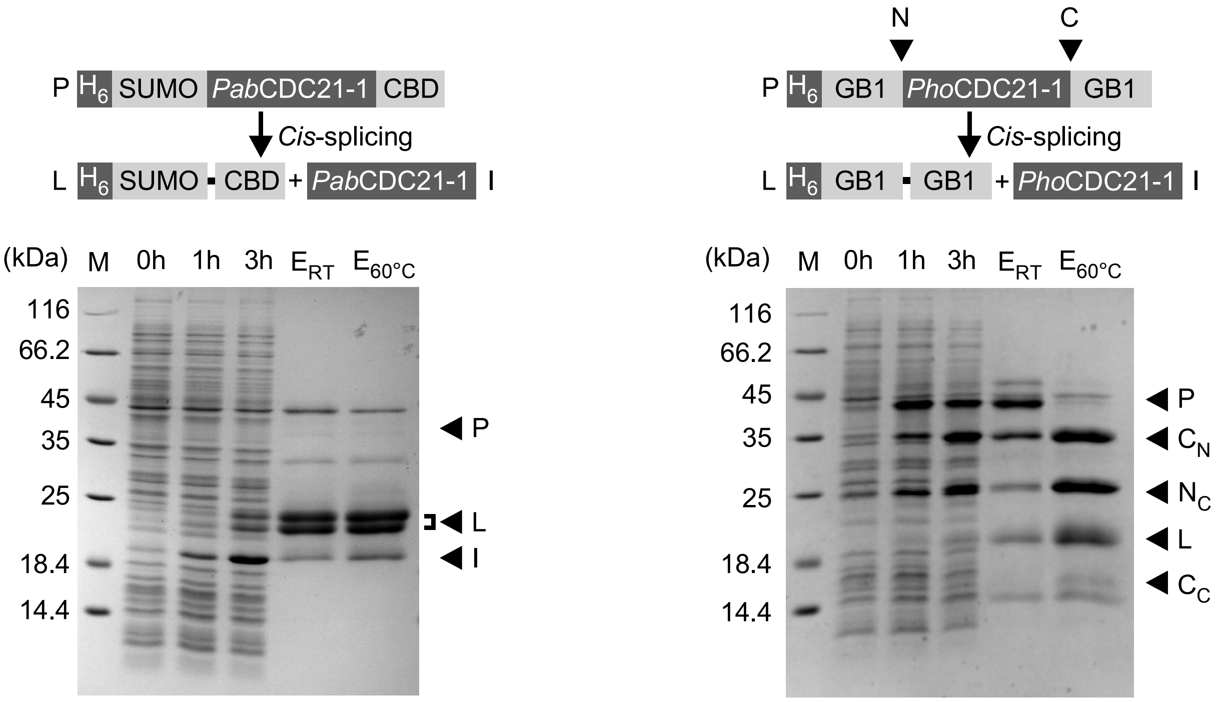

Supplementary Figure S1. *Cis*-splicing of the *Pab* and *Pho* CDC21-1 inteins *in vivo* and *in vitro*. The inteins were expressed in *E. coli* flanked by eihter a SUMO and CBD domain (*Pab*CDC21-1), or by two GB1 domains (*Pho*CDC21-1). Splicing products and educts were IMAC-purified at room temperature (RT) via the N-terminal His tag and afterwards incubated for 1 hour at 60°C. Samples were analyzed at various indicated stages by SDS-PAGE. M, 0h, 1h, and 3h stand for molecular marker and expression cultures before (0h), and 1 and 3 hours after induction. P, L, and I indicate the expected migration hights of precursors, ligated products, and excised intens, respectively. N and C show N- and C-terminal cleavage products which are further labeled as N- and C-terminal fragments resulting from the cleavage reactions by subscript indices. The SUMO domoain used as N-extein with the *Pab*CDC21-1 intein commonly migrates as a dual band on SDS-PAGE gels.
